# Efficacy and safety of imidacloprid 10 %/moxidectin 1 % spot-on formulation in the treatment of feline infection by *Capillaria aerophila*

**DOI:** 10.1007/s00436-012-3025-4

**Published:** 2012-07-11

**Authors:** Donato Traversa, Angela Di Cesare, Emanuela Di Giulio, Giuseppe Castagna, Roland Schaper, Gabriele Braun, Beate Lohr, Fabrizio Pampurini, Piermarino Milillo, Katrin Strube

**Affiliations:** 1 Department of Comparative Biomedical Sciences, University of Teramo, Teramo, Italy; 2 Ambulatorio Veterinario James Herriott, Roseto degli Abruzzi, Teramo, Italy; 3 Bayer Animal Health GmbH, Leverkusen, Germany; 4 Klifovet AG, Munich, Germany; 5 Bayer S.p.A., Milan, Italy

## Abstract

The nematode *Capillaria aerophila* (Trichuroidea, Trichuridae) affects the respiratory system of cats and other animals and occasionally of human beings. Infected cats may show bronchovesicular sounds, inflammation, sneezing, wheezing and, chronic cough and, sometimes, bronchopneumonia and respiratory failure. The present study evaluated the efficacy and safety of the antiparasitic spot-on formulation containing imidacloprid 10 %/moxidectin 1 % (Advocate®, Bayer Animal Health) in the treatment of natural feline infection with the lungworm *C. aerophila*. The efficacy of Advocate® administered once was tested on days 7 ± 1 and 11 ± 1 following treatment at day 0 and compared to faecal egg counts on days −6 ± 1 and −2 ± 1. Overall, 36 cats treated either with Advocate® (treatment group, *n* = 17 cats) or left untreated (control group, *n* = 19 cats) were included in the study. Geometric means of faecal egg counts values in eggs per gram of faeces were 124.03 prior to treatment and 0.26 posttreatment in treatment group, while 107.03 and 123.94 pre- and posttreatment in the untreated cats. Post-baseline egg counts showed a 99.79 % reduction in Advocate®-treated animals in comparison with cats which were left untreated. Also, treated cats showed no adverse events. This trial demonstrated that Advocate® spot-on formulation is safe and effective in the treatment of feline lung capillariosis caused by *C. aerophila*.

## Introduction

The antiparasitic spot-on formulation containing imidacloprid 10 %/moxidectin 1 % (Advocate®, Bayer Animal Health) is marketed for the treatment of different ecto- and endoparasites affecting cats. The neonicotinoid imidacloprid is an insecticide, while moxidectin is an endectocide macrocyclic lactone with a broad-spectrum of activity. Therefore, Advocate® can be applied dermally in cats for the effective and safe control and/or prevention of a range of major internal (i.e. hookworms, roundworms and heartworms) and external (i.e. mites, fleas) parasites. As key examples, this formulation has shown to be highly effective in the treatment of feline otocariosis (Fourie et al. 2003) and can be used safely even in cats heavily infected with adult *Dirofilaria immitis* (Arther et al. 2005). Also, a recent experimental study has demonstrated the high efficacy and safety of Advocate® for the treatment of feline respiratory infection caused by the metastrongylid lungworm *Aelurostrongylus abstrusus* (Traversa et al. 2009a).

Along with *A. abstrusus*, *Capillaria aerophila* (Trichuroidea, Trichuridae) is another nematode affecting the lungs of cats. The adult stages live embedded in the respiratory epithelium of bronchioles, bronchi and trachea of the definitive host, i.e. cats, dogs, wild carnivores and occasionally humans. The parasites damage the lung parenchyma and induce chronic bronchitis characterised by symptoms of varying severity, i.e. bronchovesicular sounds, inflammation, sneezing, wheezing, and chronic dry or moist and productive (i.e. especially when bacterial complications occur) cough; when the parasite burden is heavy, the diseases can lead to mortality due to bronchopneumonia and respiratory failure (Holmes and Kelly 1973; Bowman et al. 2002; Taylor et al. 2007; Burgess et al. 2008; Traversa et al. 2009b).

The infection caused by *C. aerophila* is still underestimated, albeit the nematode is globally distributed and apparently spreading in different countries; moreover, it may cause relevant clinical pictures and has a certain zoonotic potential (Lalosević et al. 2008; Conboy 2009; Traversa et al. 2010). In the past few years, gaps have been filled on its morphology and biology (Traversa et al. 2011), epidemiology (Traversa et al. 2009b; Di Cesare et al. 2011), molecular diagnosis (Di Cesare et al. 2012a) and genetic make-up (Di Cesare et al. 2012b). Nevertheless, information on effective and safe anthelmintic therapy of pulmonary capillariosis is almost non-existent and those scanty information published are related to few single clinical cases. Given the significant merit in establishing reliable protocols for the therapy of the infection caused by *C. aerophila*, the aim of the present study was to evaluate the efficacy and safety of Advocate® for the treatment of natural feline capillariosis by *C. aerophila*.

## Materials and methods

### Study design

The study was carried out from July 2009 to October 2011 as a controlled, randomised, multicentric field trial in accordance to Good Clinical Practice by the European Agency for the Evaluation of Medicinal Products (CVMP/VICH GL7, July 2000; CVMP/VICH GL19, July 2001).

The blinded investigator laboratory (site A) was the Parasitology Laboratory at the Department of Comparative Biomedical Sciences, University of Teramo, Italy. Seven veterinary practices in central Italy were in charge for the enrolment of the cats (see Acknowledgments). The practices were designated as site B (located in Isola del Gran Sasso Municipality, Abruzzo region), site C (Pescara Municipality, Abruzzo region), sites D and H (Ascoli Piceno Municipality, Marche region), site E (Teramo Municipality, Abruzzo region), site F (Castelnuovo Vomano Municipality, Abruzzo region) and site G (Alba Adriatica Municipality, Abruzzo region).

After a pre-inclusion selection via a qualitative faecal examination, positive animals underwent two pretreatment quantitative copromicroscopic examinations at days −6 ± 1 and −2 ± 1, a clinical examination at anthelmintic treatment (day 0) and two posttreatment quantitative copromicroscopic examinations at days 7 ± 1 and 11 ± 1.

### Pre-inclusion screening

Five hundred and forty-two cats, i.e. 107, 125, 75, 32, 101, 27 and 75 in sites B–G, respectively, were subjected to a screening examination for *C. aerophila* eggs by a qualitative copromicroscopic examination by two different floatation procedures employing a sugar solution with a 1.200 specific gravity (s.g.) and a 1.350 s.g. zinc sulphate solution (Sloss et al. 1994).

**Fig. 1:**
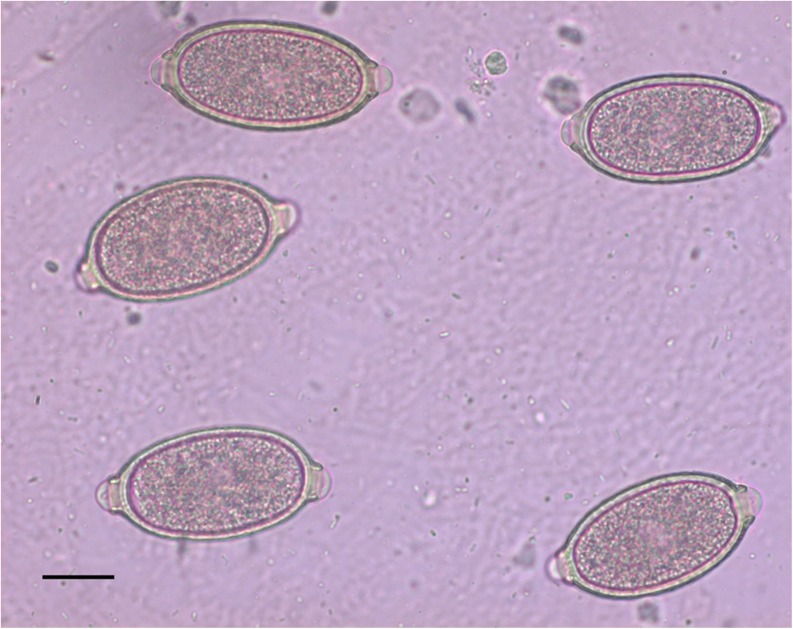
Eggs of *C. aerophila*. Scale bar, 20 μm

Eggs of *C. aerophila* (Fig. 1) were identified at the species level by the characteristic size, shape, plug morphology and appearance of the egg shell (Traversa et al. 2011). All cats with positive results for *C. aerophila* at this screening were further evaluated by inclusion and exclusion criteria for the enrolment in the study.

### Inclusion and exclusion criteria

Animals which scored positive for eggs of *C. aerophila* at the pre-inclusion were subjected to two quantitative copromicroscopic examinations with a McMaster method (Sloss et al. 1994) at days −6 ± 1 and −2 ± 1 to evaluate the pretreatment values of egg per gram of faeces (EPG). Inclusion criteria were:

- Cats positive at least at one of the above four examinations;
- Cats ageing more than 9 weeks
- Cats weighing more than 1 kg

Animals which met these criteria were enrolled with the owner's consent to participate in the study. The study was continued only if the animals were considered suitable for the study when examined physically on day 0 (treatment).

Cats could be excluded according to the following exclusion criteria:

- Negativity at the two pre-inclusion quantitative examinations;
- Cats treated within the last 2 months with any anthelmintic drug, including macrocyclic lactones;
- Pregnant or lactating queens;
- Cats with severe systemic diseases and/or with a general bad health.

### Anthelmintic treatment

Of the 542 screened cats, 36 (6.6 %) animals from 6 sites were infected and recruited. Specifically, 13 come from site B (12.1 % of cats screened in the Site), 1 from site C (0.8 %), 2 from site D (2.6 %), 6 from sites E (18.7 %) and F (5.9 %) each, and 8 from site H (10.6 %). Site G enrolled no positive animals in the study. At day 0, the animals were clinically examined prior to treatment and then allocated to two groups, one treated (treatment group) with the spot-on solution containing imidacloprid 10 %/moxidectin 1 % (Advocate®) and one left untreated (control group), as per a randomised block design in a ratio of 1:1.

Cats in the first group were treated with Advocate® spot-on solution as single dosage applied according to label instructions, while animals in the control group did not receive any compound till the end of the study. All control cats received a rescue dose of Advocate® after the completion of the study.

A complete clinical examination was performed posttreatment to evaluate safety of the treatment and potential adverse events. The occurrence of adverse events (AE) could be registered, considering definitions accordingly to the Good Clinical Practice guidelines (CVMP/VICH GL9, July 2000).

### Posttreatment evaluation

Faecal samples collected from the 36 cats at days 7 ± 1 and 11 ± 1 were subjected to two quantitative McMaster examinations. Primary efficacy criterion was the reduction of post-baseline EPG faecal egg counts. The highest value from the four egg counts performed in the pretreatment assessment (i.e. two examinations at days −6 ± 1 and −2 ± 1) was used as baseline value. From the four faecal egg counts performed posttreatment, the highest value was used for the calculation of efficacy. The analysis of the efficacy criterion was performed on the log-transformed scale using an analysis of covariance adjusted for baseline egg counts. Geometric means were calculated as: *G*_mean_ = *e* ^AML^ − 1, where AML = the arithmetic mean of the EPG counts. The difference between the geometric mean (GM) of EPG before and after treatment was determined and expressed as an efficacy value using the following formula: efficacy % = 100 × (GM pretreatment − GM posttreatment)/GM pretreatment. The percentage decrease was considered effective if efficacy was at least 90 %.

## Results

All enrolled 36 cats completed the study according to the protocol. They were appropriately treated, no cats were removed from the study subsequent to inclusion for any reason and all of them were included in efficacy calculations.

### Pretreatment egg counts

Geometric means of EPG at baseline were 124.03 (arithmetic mean 152.9 ± 128.1) in the Advocate^®^ and 107.03 (arithmetic mean 118.4 ± 53.3) in the untreated control group. No statistically significant (*P* = 0.568) differences in egg counts in pretreatment period between the two groups were found.

### Efficacy evaluation

In the treatment group, EPG value was reduced from 152.9 (±128.1) at baseline to 2.9 (±12.1) at day 7 or 11. In the untreated animals, an increase from 118.4 (±53.3) to 163.2 (±165.7) EPG was found. Geometric means of EPG were 124.03 (baseline) and 0.26 (posttreatment) in the treatment group and 107.03 (baseline) and 123.94 (posttreatment) in the control cats. Relative change from baseline was 99.79 and −15.8 % in the treated and control cats, respectively (Table 1). The difference between groups in change from baseline was 4.672 (95 % confidence interval from 4.128 to 5.216) which is statistically significant at *P* < 0.0001, thus showing superiority of Advocate^®^ spot-on compared to the untreated group.

**Table 1:** Efficacy calculation of Advocate® against *C. aerophila* infection in treatment (*n* = 17 cats) and control groups (*n* = 19 cats)

	Treatment group (Advocate®)		Control group (untreated)		
Study period and statistics	Actual	Change	Actual	Change	Difference^a^ B − A
Baseline EPG					
Mean (SD)	152.9 (128.1)		118.4 (53.3)		
95 % CL	87.1; 218.8		92.8; 144.1		
Min to max	50 to 600		50.0 to 250.0		
Median	100		100.0		
*G*_mean_	124.03		107.03		
Post-baseline EPG					
Mean (SD)	2.9 (12.1)	−150.0 (128.7)	163.2 (165.7)	44.7 (139.3)	170.98 (118.84)
95 % CL	−3.3; 9.2	−216.2; −83.8	83.3; 243	−22.4; 111.9	88.90; 253.07
Min to max	0 to 50	−600.0 to −50.0	50 to 800	−50.0 to 600	*F* = 17.9595
Median	0.0	−100.0	150.0	0.0	*P* = 0.0002
*G*_mean_	0.26	99.79 %	123.94	−15.8 %	

Maximum actual counts of faecal egg count in terms of *C. aerophila* eggs per gram of faeces (EPG) pretreatment (baseline, days −6 ± 1 and −2 ± 1) and post-baseline (days 7 ± 1 and 11 ± 1) and change from baseline are indicated. Mean and standard deviation (SD), 95 % confidence level (CL), minimum (min), maximum (max), median and geometric mean of EPG values are also reported

^a^Difference and 95 % CI form ANOVA adjusted for baseline

### Adverse events, clinical income and outcome

No AEs were recorded in any of the treated cats. Clinical symptoms were present prior to treatment (day 0) in three cats, thus were not considered to be related to the study medication. More specifically, at clinical examination, three animals showed cough due to capillariosis and a concomitant infection by *A. abstrusus* (i.e. two cats belonging to treatment group in site E), and cough due to *C. aerophila* and diarrhoea of unknown origin (i.e. one cat from untreated control group from site F).

At completion of the study, all 36 enrolled cats but one were clinically healthy. Specifically, respiratory distress disappeared in the two symptomatic cats from site E while cough was still present in the untreated cat of the control group. However, in this cat, cough disappeared after rescue treatment at study completion.

## Discussion

This study demonstrated that a single administration of Advocate® spot-on at a minimum dose per kilogram body weight of 10 mg imidacloprid and 1 mg moxidectin is safe and effective in the treatment of feline infection by *C. aerophila* under field conditions. The efficacy of this anthelmintic formulation was investigated on the basis of copromicroscopic results. Indeed, a modified quantitative McMaster method was selected to determine the pre- and posttreatment infection status (Sloss et al. 1994) of the cats recruited for the study. Even though a sensitivity <100 % cannot be ruled out, if compared with necropsy or experimental molecular assays, the use of this method still is the most reliable approach to detect *C. aerophila* eggs and, for this reason, it was chosen for the success criteria in the study. The diagnostic sensitivity of the test, and thus the reliability of the data obtained, was assured by two consecutive quantitative examinations repeated twice at each of the pre- and posttreatment sample collection. Furthermore, the identification of *C. aerophila* ova in positive samples was carefully performed with no risks of misidentification. In fact, while barrel/lemon-like eggs found in faeces of dogs require a thorough morphometric and morphological analysis to avoid misdiagnosis with other trichuroids, e.g. *Capillaria boehmi* or *Trichuris vulpis* (Traversa et al. 2011), these eggs are usually not a diagnostic problem for cats. Indeed, feline whipworms (i.e., *Trichuris felis*, *Trichuris campanula* and *Trichuris serrata*), regardless they are separate species or the same, debated, parasite (Cameron 1937; Ng and Kelly 1975; Bowman et al. 2002), are only confined in few areas of the Americas and few other regions, where they have been reported very rarely in the last 40 years (Kelly 1973; Ng and Kelly 1975; Hass and Meisels 1978; Bowman et al. 2002; Castro et al. 2009). Although morphological and morphometrical studies of feline trichuroids are few and old (Kelly 1973; Ng and Kelly 1975; Hass and Meisels 1978), the diagnosis of feline lung capillariosis is easy for the almost null possibility to find whipworm eggs in feline faeces and for the recently clarified diagnostic features of *C. aerophila* ova (Traversa et al. 2011).

At the time the study starts, there was no drug licensed with *C. aerophila* as an indication for use in cats. Additionally, information on the field efficacy of parasiticides in the treatment of the feline infection by *C. aerophila* are fragmentary and in most cases related to single clinical cases.

The repeated administration of dichlorvos was unsuccessful in treating clinical signs and stopping egg shedding in an infected cat. Then, the same cat was treated with injectable levamisole at a dosage of 7.5 mg/kg for two consecutive days, repeated as a single administration after 2 weeks. This protocol has shown effect in reducing clinical signs and egg shedding (Endres 1976). The same molecule has been used in another single case at the oral dose of 5 mg/kg for five consecutive days, administered three times, with intervals of 9 days between treatments. At day 30 after the first administration, clinical signs disappeared and at days 40 and 150 the animal scored negative upon copromicroscopic examinations (Norsworthy 1975). However, this report can be questioned by the evaluation of the therapy, stated to have relied on the detection of eggs and larvae of *C. aerophila* by a Baermann method. Actually, larval *C. aerophila* are not found in fresh faeces, given that the eggs embryonate not before 1 month after emission (Traversa et al. 2011).

With regard to macrocyclic lactones, two *off label* subcutaneous administrations of 300 μg/kg of abamectin 14 days apart have led to reduction of cough and nasal discharge in an infected cat, which also became negative at the faecal examination (Barrs et al. 2000). The efficacy of ivermectin against different endoparasites has been evaluated in a study carried out in 24 naturally infected cats. Two of these animals, which were treated orally with ivermectin either at 100 or 300 μg/kg, scored copromicroscopically positive for *Capillaria* spp. after treatment with the former and negative at copromicroscopy and necropsy with the latter; however, these data were stated to be too limited to infer any conclusion on the efficacy of ivermectin against *Capillaria* spp. (Blagburn et al. 1987).

The present study fills an important gap in treatment options for lung capillariosis in cats, in that the macrocyclic lactone moxidectin present in the Advocate® spot-on formulation proved to be effective against *C. aerophila* in a number of infected cats. In general, the single application of Advocate® at the recommended dosage leads to a high moxidectin concentration and long elimination half-life, which provide a prolonged activity against major feline parasites. After the recent experimental demonstration that Advocate® is effective against the major cat lungworm *A. abstrusus* (Traversa et al. 2009a), the present study allows to include another respiratory nematode affecting cats, *C. aerophila*, among the feline parasitic nematodes that can be effectively and safely treated by this spot-on.

For its safety and efficacy, Advocate® can be considered a suitable choice for the treatment of cat lung capillariosis. Also, this formulation overcomes the constraints of parasiticides with a certain efficacy against *C. aerophila*, which have been applied off label and/or in oral or parenteral formulations even in repeated dosages. In fact, the use of oral anthelmintics in feline clinical practice is often impaired by difficulties of the administration to indocile or feral animals and the need of multiple dosing. Indeed, Advocate® has advantages in the current practice for the treatment of cat capillariosis, i.e. the possibility of a single dose and the easy-to-apply spot-on administration.
